# Emerging applications of phage therapy and fecal virome transplantation for treatment of *Clostridioides difficile* infection: challenges and perspectives

**DOI:** 10.1186/s13099-023-00550-3

**Published:** 2023-05-09

**Authors:** Hamideh Raeisi, Maryam Noori, Masoumeh Azimirad, Seyed Reza Mohebbi, Hamid Asadzadeh Aghdaei, Abbas Yadegar, Mohammad Reza Zali

**Affiliations:** 1 Foodborne and Waterborne Diseases Research Center, Research Institute for Gastroenterology and Liver Diseases, Shahid Beheshti University of Medical Sciences, Tehran, Iran; 2 Gastroenterology and Liver Diseases Research Center, Research Institute for Gastroenterology and Liver Diseases, Shahid Beheshti University of Medical Sciences, Tehran, Iran; 3 Basic and Molecular Epidemiology of Gastrointestinal Disorders Research Center, Research Institute for Gastroenterology and Liver Diseases, Shahid Beheshti University of Medical Sciences, Tehran, Iran

**Keywords:** *Clostridioides difficile*, Phages, Phage therapy, Gut microbiome, Fecal microbiota transplantation, Fecal virome transplantation

## Abstract

*Clostridioides difficile*, which causes life-threatening diarrheal disease, is considered an urgent threat to healthcare setting worldwide. The current standards of care solely rely on conventional antibiotic treatment, however, there is a risk of promoting recurrent *C. difficile* infection (rCDI) because of the emergence of antibiotic-resistant strains. Globally, the alarming spread of antibiotic-resistant strains of *C. difficile* has resulted in a quest for alternative therapeutics. The use of fecal microbiota transplantation (FMT), which involves direct infusion of fecal suspension from a healthy donor into a diseased recipient, has been approved as a highly efficient therapeutic option for patients with rCDI. Bacteriophages or phages are a group of viruses that can infect and destroy bacterial hosts, and are recognized as the dominant viral component of the human gut microbiome. Accumulating data has demonstrated that phages play a vital role in microbial balance of the human gut microbiome. Recently, phage therapy and fecal virome transplantation (FVT) have been introduced as promising alternatives for the treatment of *C. difficile -*related infections, in particular drug-resistant CDI. Herein, we review the latest updates on *C. difficile*- specific phages, and phage-mediated treatments, and highlight the current and future prospects of phage therapy in the management of CDI.

## Background

*Clostridioides difficile* (*C. difficile*) is a strictly anaerobic, Gram-positive, spore-forming bacillus found widely in the mammalian gastrointestinal (GI) tract [1]. *C. difficile* can infect the human colon and cause mild to severe diarrhea, particularly nosocomial-associated diarrhea, which is considered as a serious threat to both public health and healthcare setting worldwide [2]. Other common clinical manifestations of *C. difficile* infection (CDI) include abdominal pain and distention, colon inflammation, fever, leukocytosis, and tachycardia [1]. Patients with severe CDI are at a great risk for the development of pseudomembranous colitis (PMC), toxic megacolon, bowel perforation, sepsis, and even death [3]. Furthermore, elderly hospitalized patients (> 65 years) under antibiotic therapy are considered as the population-at-risk for CDI development, however, the incidence and severity of CDI in the community have also globally increased in the last two decades [4, 5]. The mortality rate of CDI has been reported to range from 2% of all deaths to more than 20% of CDI-attributable mortality, and the overall mortality rate was estimated to be 22% [6]. Proper antibiotic therapy, including vancomycin and fidaxomicin, is recommended as the first-line treatment for CDI [2]. Although antibiotic therapy is currently the reasonably effective treatment option for CDI, its long-term use may lead to gut microbiota dysbiosis (according to relative abundance and diversity), reduced susceptibility of *C. difficile* against antimicrobial agents, and the emergence of antibiotic-resistant and hypervirulent *C. difficile* strains [1, 7, 8]. Additionally, antibiotic therapy may perpetuate the risk of recurrence and increase the vulnerability of CDI patients to *C. difficile* re-colonization for about 2 to 6 weeks following completion of a course of therapy [9, 10].

The worrying side effects of antibiotics have persuaded the researchers to explore novel complementary and alternative therapeutic strategies such as antibody therapy [11], fecal microbiota transplantation (FMT) [12], fecal virome transplantation (FVT) [13], and phage therapy [14] for the treatment, prevention, and reducing the rate of rCDI. FMT is a safe and feasible alternative to antibiotic therapy with high cure rates, which has been suggested for treating refractory CDI by the latest American College of Gastroenterology (ACG) clinical guidelines [15, 16]. It has been established that FMT leads to restoration of the gut microbiota, however, its precise mechanism of action is not fully elucidated [12]. In addition to the gut bacteria, the diversity and composition of gut viral community (virome) are also modified through FMT [17]. Recent evidence showed that bacteriophages can play an essential role in successful treatment and outcome of FMT by manipulating bacterial communities [17]. Recently, the use of phage-mediated treatments has attracted much attention as a promising target and/or tool for treating human microbial infections. Accordingly, recent studies have demonstrated that FMT, FVT, or phage therapy can cause gut virome restoration with high efficiency in several clinical indications such as obesity, infectious diseases, and particularly rCDI [13, 17–19]. Notably, the application of phage-based treatments, including FVT and phage therapy, could offer beneficial advantages in clinical experience compared with current microbiome-related therapeutics, including the use of antibiotics, bacterial probiotics, and even FMT, which may further lead to bacterial microbiome distortion and cause gut dysbiosis [14]. In this work, we review the significance of the gut phageome in the pathogenesis of *C. difficile*. Moreover, we summarize the current approaches of phage therapy used for treating CDI and discuss its present limitations and prospective, providing exciting opportunities for virome-based therapeutics against CDI.

## An overview of bacteriophages

**Fig. 1:**
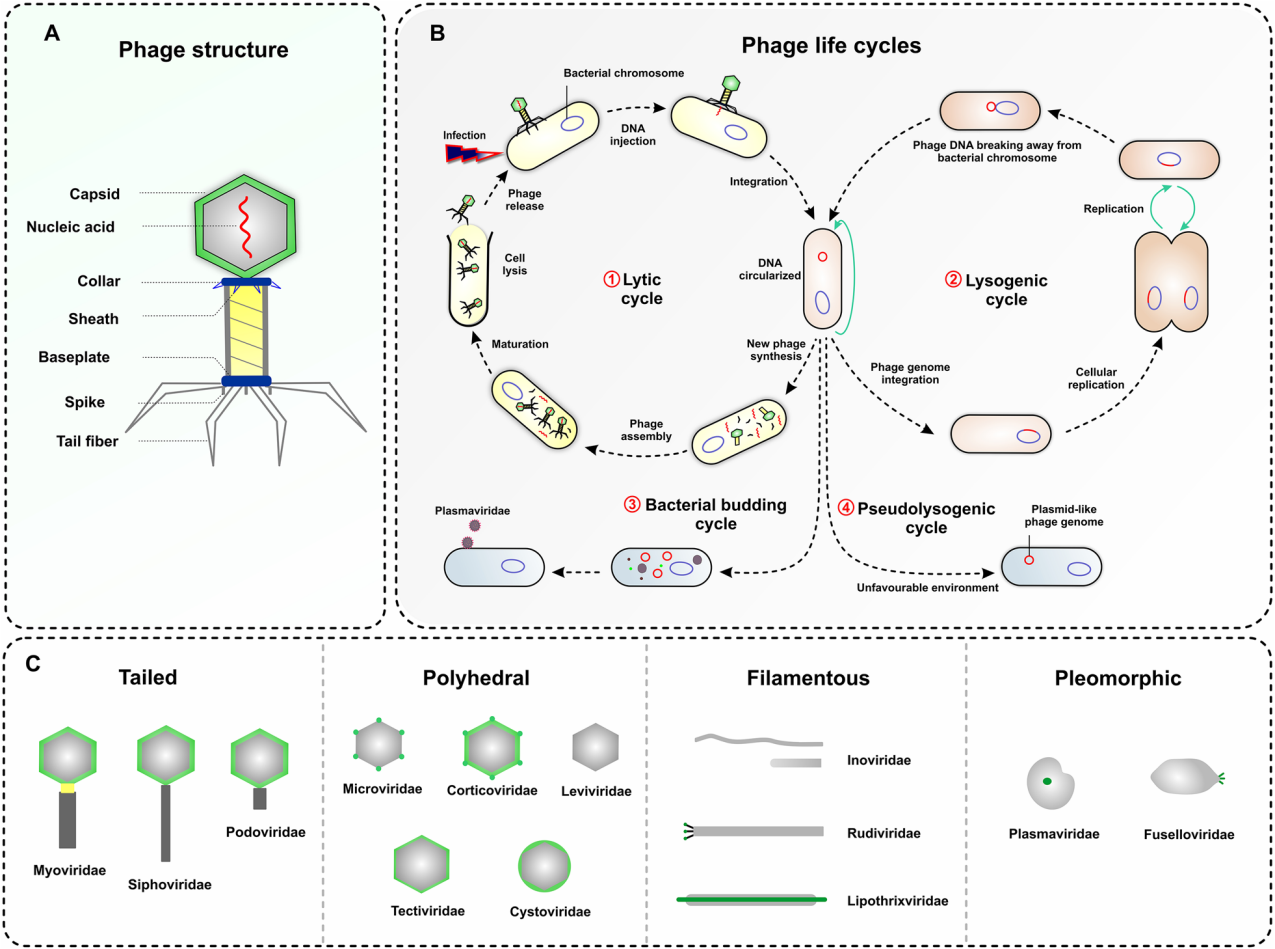
An overview on structure, morphology, and life cycles of bacteriophages. **A** A schematic of typical bacteriophage structure (T4). **B** A diagram illustrating life cycles of bacteriophages which starts with the attachment of phage particle to the cell surface and followed by phage genome insertion. Phages can apply four life cycles during infection: (1) in lytic cycle, phage genome is replicated, new virions are synthesized and released through the bacterial cell lysis; (2) in lysogenic cycle, phage genome is integrated into the bacterial genome or in a plasmid-like construct (episomal state) as a prophage, and replicated with the bacterial chromosome as long as bacteria divide. The prophage remains in a dormant state in the infected bacteria unless encountering a stimulating factor, such as antibiotics, ultraviolet radiation, temperature or pH alterations, which may cause resuming a lytic cycle; (3) in bacterial budding cycle, phages can bud out of bacterial cells and protect the host cell from lysis or death; (4) in pseudolysogenic cycle, the phage genome remains as an episomal in the host cell without integration into the host genome or replication. **C** Representation of bacteriophage morphotypes including tailed, polyhedral, filamentous, and pleomorphic phages, following with some examples for each morphotype

Bacteriophages (phages), the most diverse and abundant biological entities on the planet, are viruses that specifically infect bacteria for reproduction [20]. Structurally, most phages consist of a viral genome packaged in coat protein (called the capsid) [21]. In addition to capsid proteins, some phages present an outer lipid membrane or lipoprotein envelope (Fig. 1A) [20]. The phage morphology is highly variable and can be tailed, polyhedral, pleomorphic, or filamentous [22]. Phages are also variable in genome size, which can range from very simple (~ 3.5 kb) to highly complex (∼540 kb), and are composed of either single-stranded DNA (ssDNA), double-stranded DNA (dsDNA), ssRNA, or dsRNA [21]. Notably, phage genomes evolve rapidly by horizontal gene transfer (HGT) with the genetic materials of host and other phages, resulting in typically mosaic genomes [23]. Genome mosaicism is described as the genetic heterogeneity, which is specified by highly similar sequences interspersed with sequences with no significant similarity [24, 25]. The genome mosaicism of phages significantly complicates the taxonomic classification; however, phages are classified according to the type of their nucleic acid and structural conformation [21]. Generally, the International Committee for Taxonomy of Viruses (ICTV) classified bacteriophages into 19 families [26], which are summarized in Table 1.

**Table 1:** Overview of classification and basic properties of bacteriophages

Family	Nucleic acid	Particulars	Symmetry	Genome size (kb)
*Myoviridae*	dsDNA	Contractile tail, non-enveloped	Binary	34–169
*Siphoviridae*	dsDNA	Long non-contractile tail, non-enveloped	Binary	34–169
*Podoviridae*	dsDNA	Short non-contractile, non-enveloped tail	Binary	34–169
*Tectiviridaea*	Linear, dsDNA	Isometric, non-enveloped	Cubic	15
*Corticoviridae*	Circular, dsDNA	Isometric, non-enveloped	Cubic	10
*Lipothrixviridae*	Linear, dsDNA	Rod-shaped, enveloped	Helical	16–42
*Plasmaviridae*	Circular, dsDNA	Pleomorphic, enveloped	Helical	12
*Rudiviridae*	Linear, dsDNA	Rod-shaped, enveloped	Helical	32–35
*Fuselloviridae*	Circular, dsDNA	Lemon shaped, non-enveloped	Pleomorphic	15–18
*Inoviridae*	Circular, ssDNA	Filamentous, non-enveloped	Helical	5–9
*Microviridae*	Circular, ssDNA	Isometric, non-enveloped	Cubic	4–6
*Leviviridae*	Linear, ssRNA	Isometric, non-enveloped	Cubic	3–4
*Cytoviridae*	Linear, dsRNA	Spherical, enveloped	Cubic	13
*Ampullaviridae*	Linear, dsDNA	Bottle-shaped, enveloped	Helical	14–17
*Bicaudaviridae*	Circular, dsDNA	Lemon-shaped, non-enveloped	Helical	80–100
*Clavaviridae*	Circular, dsDNA	Rod-shaped, non-enveloped	Helical	5278
*Globuloviridae*	Linear, dsDNA	Isometric, enveloped	Helical	20–30
*Guttavirus*	Circular, dsDNA	Ovoid, non-enveloped	Pleomorphic	15–75
*Inoviridae*	Circular, dsDNA	Filamentous, non-enveloped	Helical	5.5–10.6

*dsDNA* double-stranded DNA, *ssDNA* single-stranded DNA, *ssRNA* single-stranded RNA, *dsRNA* double-stranded RNA

Bacteriophages can significantly affect the evolution of the gut microbiota [27]. The ability of bacteriophages to alter gut microbiota composition mainly depends on their replication cycles, which are also known as life cycles. In general, bacteriophages exhibit four life cycles, including lytic, lysogenic, bacterial budding, and pseudolysogenic cycles (Fig. 1B) [28, 29]. In the lytic cycle, the phage injects its genome into the host cell cytoplasm and produces new phage particles within 30–60 min by using the bacterial cell machinery. At the end of each cycle, the infected cell is lysed and 20–200 new phage particles are released [21, 23]. The new phage particles can infect other susceptible host cells in the vicinity. The lytic phage phenotype is pervasive under critical biological events such as environmental stress and gut inflammation that potently regulate the composition of the gut bacterium [30, 31]. Lysogenic phages, also known as temperate phages, employ the lysogenic cycle. This life cycle is obviously different from the lytic cycle. Temperate phages are able to integrate their genome into the chromosome of their host bacteria as a prophage, or remains as a stable extra-chromosomal genetic element, instead of immediately producing new phage particles. This life cycle helps temperate phages to profit from the survival of their host bacteria in unfavorable environmental conditions [29]. Virulent phages exploit exclusively the lytic life cycle, whereas temperate phages can benefit both the lytic and lysogenic pathways [32]. Therefore, the prophage form can be advantageous to the survival or proliferation of a temperate phage when faced with different external challenges [30]. Generally, the prophage induction is both phage - and inducer-dependent and can be triggered by harsh environmental conditions such as nutrient limitation, phagocytosis, antibiotics, alterations in pH and temperature, ultraviolet radiation, chemical/diet inducers, oxidative/inflammatory stressors, or even superinfection of the host cell by other strains of phages [29, 33, 34]. Recently, short-chain fatty acids (SCFAs) and fructose-enriched diets were added to the list of prophage inducers in bacteria, particularly lactobacilli [35]. Notably, switching the life cycle of phages from lysogenic to lytic under favorable conditions can alter the composition of the gut microbiota [36]. Additionally, some phages can initiate a pseudolysogenic life cycle under harsh environmental challenges. In this life cycle, the phage genome remains as a plasmid-like construct (episomal) in the host cell without integration into the host genome or replication [37]. Occasionally, some phages, e.g., *Plasmaviridae*, can apply a special replication cycle, which bud out of the bacterial cells, and thus protect the host cell from lysis or death [38].

## Effects of phage community on gut microbiota structure

The human gut virome is dominated by bacteriophages, which are important resident players in the human gut and can affect gut microbiota structure [36]. They can strongly impact the composition and function of bacterial communities, physiology, evolution, and population dynamics by increasing the functional diversity of bacteria or phage communities, and help maintain gut homeostasis [17, 36]. However, a dysbiotic gut virome can boost an imbalance (dysbiosis) in the composition of the gut microbiota through changing bacterial richness and diversity [39].

Recently, several human diseases including inflammatory bowel disease (IBD), colorectal cancer, rCDI, severe acute respiratory syndrome coronavirus 2 (SARS-CoV-2) infection, diabetes, obesity, and malnutrition have been linked to an imbalanced gut virome [40–44]. Whether alteration in the composition of viral communities is a cause or a consequence of the disease, is thought-provoking and warrants profound and comprehensive investigations [39]. Depending on the conditions, phages can adapt their life cycle typically to either lytic or lysogenic cycles [21]. The lytic phages can benefit from predator-prey relationships with their host, leading to altering the functional composition of microbial communities [29]. Therefore, virulent phages can exert detrimental impacts on their bacterial host cells via cell lysis, and consequently balance the overall population of the gut microbiota [45]. Interestingly, the lysogenic cycle of phages can provide opportunities to their hosts at a community level. Some intestinal phages, e.g., filamentous phages, can promote environmental compatibility by regulating the viability and metabolism of bacterial hosts, and influence their virulence via regulating the expression of the genes involved in biofilm formation and exogenous toxin production [40, 46, 47]. Importantly, it has been documented that toxin-encoding genes of a number of bacterial pathogens are carried by prophage genome, such as Shiga-toxin of *Escherichia coli*, botulinum neurotoxins of *Clostridium botulinum*, diphtheria toxin of *Corynebacterium diphtheria*, and cholera toxin of *Vibrio cholera* [48–51]. Additionally, some studies have suggested that prophages may affect the expression of toxin A (TcdA) and toxin B (TcdB) of *C. difficile* bacteria [52, 53]. Notably, phages together with other mobile genetic elements (MGEs) can cause HGT across bacterial communities during the lysogenic process, which may help improve the environmental adaptability of bacterial pathogens through acquisition of new traits related to survival, virulence, pH tolerance, or antibiotic resistance [46, 52, 54, 55]. Accordingly, the acquisition of antibiotic-resistant genes (ARGs) by phage transduction has been reported in some infectious bacteria, such as *C. difficile*, *Staphylococcus aureus*, and *Enterococcus faecalis* [56–58]. In contrast, some studies have shown that human-associated phageome rarely carries ARGs, suggesting that some ARGs are unrealistically attributed to phages [59, 60].

Additionally, it is well established that various factors encoded by phages can strengthen bacterial pathogenicity by influencing their virulence attributes such as adhesion, invasion, colonization, and toxin production [49, 61]. For example, a protein encoded by phages, ankyrin protein (ANKp), is able to attenuate the innate immunity of endothelial cells against *E. coli*, leading to development of infection [62]. Environmental stress may affect bacterial mortality for optimizing energy usage and cause phages to switch from lysogeny to lytic cycles [63]. The remains of lysed cells provide nutrients for survivors and the possibility of transduction of the remained genes into survivors by HGT, thus, helping increase the functional efficiency of the community [64]. It seems that mechanisms of the action of phages can exert an evolutionary effect on their host, due to the genetic exchange between bacteria during repeated cycles of phage infection or lysogenic conversion [65, 66]. All in all, it can be concluded that interactions between bacteriophages and their host can influence the composition of bacterial communities, which may contribute to disease development or suppression [14].

### ***C. difficile*phages**

Most *C. difficile* strains carry a set of different prophages inside their genomes [46]. These prophage-related elements have narrow host ranges in various bacterial species and can infect different *C. difficile* strains [67]. Typically, all known infecting phages of *C. difficile* belong to temperate families, including *Myoviridae* and *Siphoviridae* of *Caudovirales* order [68], and carry genomes of approximately 31–56 kb in length and a GC content of about 28–30% (Table 2) [69]. The interaction between phages and *C. difficile* strains depends on the availability of suitable receptors on bacterial host cells. There is little evidence about the phage receptors on the cell surface of *C. difficile*. So far, some studies have shown that surface layer (S-layer) protein, SlpA, may be a phage receptor candidate for *C. difficile* phages [70, 71]. Recently, Whittle et al., supported this claim and showed that SlpA of *C. difficile* can act as a cell surface phage receptor [72]. Each *C. difficile* strain contains phage-related genomic regions and carries 1 to 6 prophages. Some prophages have a large genome (> 130 kb), which can be stable as extrachromosomal DNA in *C. difficile* cells [67]. Hence, *C. difficile* genome is typically mobile and mosaic, for example, 11% of the genome of *C. difficile* strain 630 has been originated from MGEs [73]. Additionally, there are some reports about the diversity of prophages in different clinical *C. difficile* ribotypes (RTs). Based on these results, almost all discovered phages belong to myovirus, some RTs carry siphovirus prophages, and few RTs are positive for dual phage type carriage including myophages and siphophages [67, 74]. These reports have supported the coexistence of prophages with *C. difficile* through integration into its genome. These genetic exchanges may improve the bacterial adaptation in the GI tract by the acquisition of new traits [75]. However, *C. difficile* strains possess efficient defense systems to balance genetic gain and diversity, by which they can survive within phage-rich gut communities, and avoid over-acquisition of foreign genetic elements such as phages and plasmids [76]. Presently, the mechanisms of action for some of the *C. difficile* anti-phage defense systems are elucidated. These systems can be activated after injection of phage DNA, such as restriction-modification systems, and clustered regularly interspaced short palindromic repeats/CRISPR-associated protein 9 (CRISPR/Cas9), leading to inactivation of infection by breaking phage DNA [68, 76, 77]. Additionally, bacterial defense systems can prevent infection spread through toxin-antitoxin systems, which actuate a suicidal host response or dormancy [32]. Interestingly, endogenous prophages may express proteins, which block phage binding to bacterial surface receptors or restrict DNA injection, thus, preventing superinfection of their host [78].

**Table 2:** Characteristics of the bacteriophages isolated from *C. difficile*

Authors	Year	Phage	Bacteriophage family	*C. difficile strains*	Isolation method	Genome size (kb)	G + C (%)	Growth cycle and additional information	References
Goh et al.	2005	phiC2, phiC5, phiC8	*Myoviridae*	CD242, CD578	Induction (mytomycin C)	43.3–54.5	45.9–56.5	Lysogenic, upregulation of PaLoc: *tcdB*	[79]
		phiC6	*Siphoviridae*	CD371	Induction (mytomycin C)	36.3	36.3	Lysogenic	
Sebaihia et al.	2006	φCD630	*Myoviridae*	CD630	Induction (mytomycin C)	56.5	29.1	Lysogenic, applied for CRISPR arrays	[73]
Govind et al.	2006	φCD119	*Myoviridae*	CD602	Induction (mytomycin C)	53	28.7	Lysogenic, downregulation of PaLoc: *tcdA*, *tcdB*, *tcdR*, *tcdE*, *tcdC*	[80]
Fortier and Moineau	2007	φCD5	*Myoviridae*	CD630, CD44, CD52	Induction (mytomycin C)	NA	NA	Lysogenic	[81]
		φCD8-1, φCD8-2	*Siphoviridae*	CD630, CD44, CD52	Induction (mytomycin C)	NA	NA	Lysogenic	
Goh et al.	2007	φC2	*Myoviridae*	CD242, CD578, CD371, CD371	Induction (mytomycin C)	56	28.7	Lysogenic	[82]
Mayer et al.	2008	φCD27	*Myoviridae*	NA	Induction (mytomycin C)	50	29.4	Lysogenic, downregulation of PaLoc: *tcdA*, *tcdB*	[83]
Horgan et al.	2010	φCD6356, φCD6365	*Siphoviridae*	D38-2	Induction (mytomycin C)	37.6	28.4	Lysogenic	[84]
Sekulovic et al.	2011	φCD52	*Myoviridae*	NA	Induction (mytomycin C)	NA	NA	Lysogenic	[85]
		φCD24, φCD38-1, φCD38-2	*Siphoviridae*	CD38	Induction (mytomycin C)	41.1	30.8	Lysogenic, increase in the production of TcdB and TcdA and downregulation of metabolism like fructose and sorbitol	
Meessen-Pinard et al.	2012	φMMP01, φMMP03, φMMP04, φCD418	*Myoviridae*	CD343, CD368	Natural induction	23–51	31.6–48.4	Lysogenic	[86]
Sekulovic et al.	2014	phiCD146	*Siphoviridae*	CD146	Induction (mytomycin C)	30–60	NA	Lysogenic	[53]
Nale et al.	2016	phiCDHM1, phiCDHM2, phiCDHM3, phiCDHM4, phiCDHM5, phiCDHM6	*Myoviridae*	CD105HE1	Enrichment and induction	NA	NA	Lysogenic	[74]
		phiCDHS1	*Myoviridae*	CD105LC1	Enrichment	NA	NA	Lytic	
Rashid et al.	2016	CDKM15, CDKM9	*Myoviridae*	NA	NA	~ 50	28.98	Lysogenic	[87, 88]
Riedel et al.	2017	phiSemix9P1	*Myoviridae*	Semix9	Induction (mytomycin C)	56	26.89	Lytic	[88]
Ramirez et al.	2018	phiCD5763, phiCD5774, phiCD2955	*Siphoviridae*	LIBA-5763, LIBA-5774, LIBA-2955	Induction (mytomycin C)	131.6–134	~ 26	Lysogenic	[89]
Garneau et al.	2018	phiCD211, phiCDIF1296T	*Siphoviridae*	DSM1296 T/ATCC9689/CD211	Induction (mytomycin C)	131	26.4	Lysogenic	[90]
Phothichaisri et al.	2018	phiHN10, phiHN16-1, phiHN16-2, phiHN50	*Myoviridae*	HN10, HN16, HN50	Induction (mytomycin C)	NA	NA	Lysogenic	[70]
		φHR24, φHN10, φHN16-2, φHN50	*Myoviridae*	HN21	Induction (mytomycin C)	NA	NA	Lysogenic	
		φHN16-1	*Tectiviridae*	NA	Induction (mytomycin C)	NA	NA	Lysogenic	
Li et al.	2020	JD032	*Myoviridae*	TW69	Induction (mytomycin C)	35	29.93	Lysogenic-lytic, altering the expression of cell surface proteins	[91]
Hinc et al.	2021	phiCDKH01	*Siphoviridae*	CD34-Sr	Induction (mytomycin C)	45	28.7	Lysogenic	[92]
Whittle et al.	2022	UCD08011, UCD418, UCD1801, UCD2301	*Myoviridae*	RT078	Enrichment and induction	31–53	28.8–29.8	Lysogenic	[72]

*NA* not available

### **Outcome of phage interactions with*C. difficile***

*C. difficile* phages are involved in the development of susceptibility/virulence-associated phenotypes of their bacterial host. Some studies have demonstrated that prophages can influence the genes related to the pathogenicity of *C. difficile* and contribute to emergence of more virulent strains [46]. The most important impact of phages on the pathogenesis of *C. difficile* is their negative or positive effect on toxin expression (e.g. prophage phiSemix9P1 isolated from some *C. difficile* strains carries a locus encoding binary toxin (CDT)) [88]. Moreover, phiCD119 prophage can express RepR regulator that binds to TcdR promoter, leading to the repression of TcdR expression [93]. TcdR is an alternative sigma factor, which is involved in *tcdA* and *tcdB* expression by recruiting RNA polymerase to their promoters [94]. Thus, the expression of the phiCD119 RepR protein in *C. difficile* results in decreased toxin production. Notably, some prophages can show common features with the pathogenicity locus (PaLoc) of *C. difficile* strains. Accordingly, the PaLoc of some *C. difficile* strains can encode a phage-like holing (TcdE), which is a membrane protein and can generate pores in bacterial cell membrane and degrade the cell wall to facilitate bacteriophage release from cytoplasm [95]. Some studies have also reported that *C. difficile* phages can influence the expression of TcdA and TcdB [53, 93]. For example, infection of *C. difficile* CD274 with a temperate siphophage, called CD38-2, leads to over-expression of TcdA and TcdB up to 2-fold, indicating the role of phage in production of *C. difficile* toxins [85]. *C. difficile* phages possibly can influence toxin production either by increasing the transcription of PaLoc genes or introducing novel regulatory genes into their hosts’ genomes during lysogenic cycles [46, 68]. Encoded genes by *C. difficile* prophages could also impact the regulatory genes involved in the expression of surface proteins, quorum sensing (QS), and antibiotic resistance [52, 96]. For example, phiCDHM1 and related prophages carry an accessory gene regulator (Agr)- like QS gene, which influences phenotypes associated with *C. difficile* virulence, such as biofilm formation, oxidative resistance, and motility [74, 97, 98]. Additionally, some phages can encode a class of enzymes, known as adenosine-diphosphate-ribosyltransferases (ADPRTs), which can increase the adherence and mucosal colonization of *C. difficile* in the host mucosa [40, 99]. It has been also reported that phages can affect the expression of *C. difficile* cell wall proteins. In this regard, a recent study demonstrated that infection of *C. difficile* RT027 with phage CD38-2 causes 20-fold upregulation in the expression of cell wall protein CwpV [100]. Furthermore, infection with CD38-2 leads to the downregulation of genes associated with the uptake and metabolism of carbohydrates e.g. glucose, fructose and D-glucitol, in bacterial host cells [101]. Phage infection can also impact the regulation of bacterial defense systems. Another study demonstrated that infection of *C. difficile* RT078 with phage JD032 can change the expression of genes encoding for DNA and RNA synthesis, and suppress anti-phage systems, including toxin-antitoxin, restriction-modification, and CRISPR-Cas systems [91]. In contrast, an in vitro study demonstrated that phage øCD27 can reduce cell numbers of *C. difficile* and its toxin production without major effects on the composition of the gut microbiota [102]. Additionally, infection with lytic phages like CDHS-1 can decrease colonization and have negative effects on bacterial pathogenicity [100]. Therefore, lytic phages could be a valuable choice for therapeutic purposes against CDI.

## Phage-based treatments

### Phage therapy

The alarming rate of antimicrobial resistance has necessitated sustained research and an urgent need for new and effective alternative treatment approaches to traditional antibiotic therapy [103]. One option could be the use of phages as therapeutic agents, which can be technically effective to prevent the challenge of antibiotic resistance [14]. The application of phages to control infectious diseases in animals has been of great interest for many years. Shortly after the discovery of phages in 1915, the use of phage therapy to treat bacterial dysentery was proposed by Felix d’Herelle [19]. This hypothesis inspired the application of phages as a therapeutic tool to control bacterial infections and led to commercial production of phages in several countries until the 1940 s. However, there were some limitations in approval processes of phage-based products due to the lack of complete characterization of phages [61]. In recent decades, the increasing rise in the rate of antibiotic resistance has led to revisiting phage therapy as drug candidates. Therefore, a guideline has been proposed for the data collection on phages, which can incorporate provisions of the Food and Drug Administration (FDA) to receive approval for phages as possible therapeutics [104]. According to this guideline, an appropriate phage for therapeutic use should meet certain criteria, including having a narrow specificity range to attack specific target cells, preventing undesired lysis of commensal microbiota, the ability to replicate inside their host by hijacking host DNA replication machinery, the ability to evolve in response to host evolution, the ability to overcome some mechanisms of phage resistance in bacteria, and the inability to attack mammalian cells or having no unfavorable immune reactions [105–107]. Additionally, the choice is limited to obligate lytic phages that do not encode any virulence factor-associated genes (e.g. toxin genes or antibiotic resistance determinants) [104]. In this regard, a phage cocktail containing LH01-Myoviridae, LL5-Siphoviridae, T4D-Myoviridae, and LL12-Myoviridae (PreforPro®) has been recently introduced as a next-generation prebiotic, which colonizes common probiotic strains such as *Lactococcus*, *Bifidobacterium*, *Lactobacillus*, and *Bacillus subtilis* and enhances their efficiency through reducing the incidence and severity of GI distress [108]. Currently, all of the natural phages selected for therapeutic purposes belong to the order *Caudovirales* [26], which have exhibited desirable efficacy in controlling infectious bacteria such as *Pseudomonas*, *E. coli*, and *Salmonella enteritidis* [109–112].

So far, different strategies have been proposed for enhancing the efficacy of phage therapy such as the use of phage cocktails, combination of phage and antibiotics, phage-derived enzymes, and phage engineering [113]. Phage cocktail is introduced as an alternative to single-phage therapy, which can overcome the limitations of single-phage therapy and delay the development of bacterial resistance to phages. This method can bypass shortcoming of the narrow phage lysis spectrum and be used to target single or multiple bacterial pathogens [114, 115]. The use of phage-derived enzymes has been also considered in some studies, among them, phage lysins have been applied for the control of several bacteria [116, 117]. Phage lysins are a class of peptidoglycan hydrolases, which degrade the bacterial cell wall peptidoglycan [116]. These enzymes are safe and species-specific, and therefore do not damage the normal intestinal microbiome. Moreover, the use of phage-derived products (endolysins, phage tail-like particles (PTLPs), and fusion proteins) can reduce the possibility of the emergence of resistant pathogens [116, 118]. Moreover, combination of antibiotics with phage or phage-derived enzymes could show better therapeutic effects than single-phage therapy. Interestingly, some antibiotics can exhibit a synergistic effect on phage therapy through increasing the propagation of lytic phage in bacterial host, leading to acceleration of bacterial cell lysis and the release of progeny phages [55].

Another solution for enhancing the efficacy of phage therapy is the use of phage genetic engineering. Phage engineering can improve the therapeutic effects of phage therapy by expanding lysis spectrum of phages and inhibiting the emergence of resistant bacteria. The use of engineered genes encoding receptor-binding proteins (RBPs) in spikes and the use of CRISPR-Cas systems are the most common methods applied for phage engineering [113, 119]. The genetic engineering of RBPs is a powerful tool to produce broad-spectrum phages [119], whereas CRISPR-Cas systems integrate the short fragments of the phage genome into the CRISPR array (namely crRNAs), which leads to the production of complementary RNA sequences. The crRNAs guide the Cas protein complex for targeting or depredating specific foreign genetic elements [113, 120]. To sum up, these strategies can improve phage therapy outcomes and has a great prospect for preventing or treating drug-resistant bacterial infections.

### Fecal virome transplantation

**Fig. 2:**
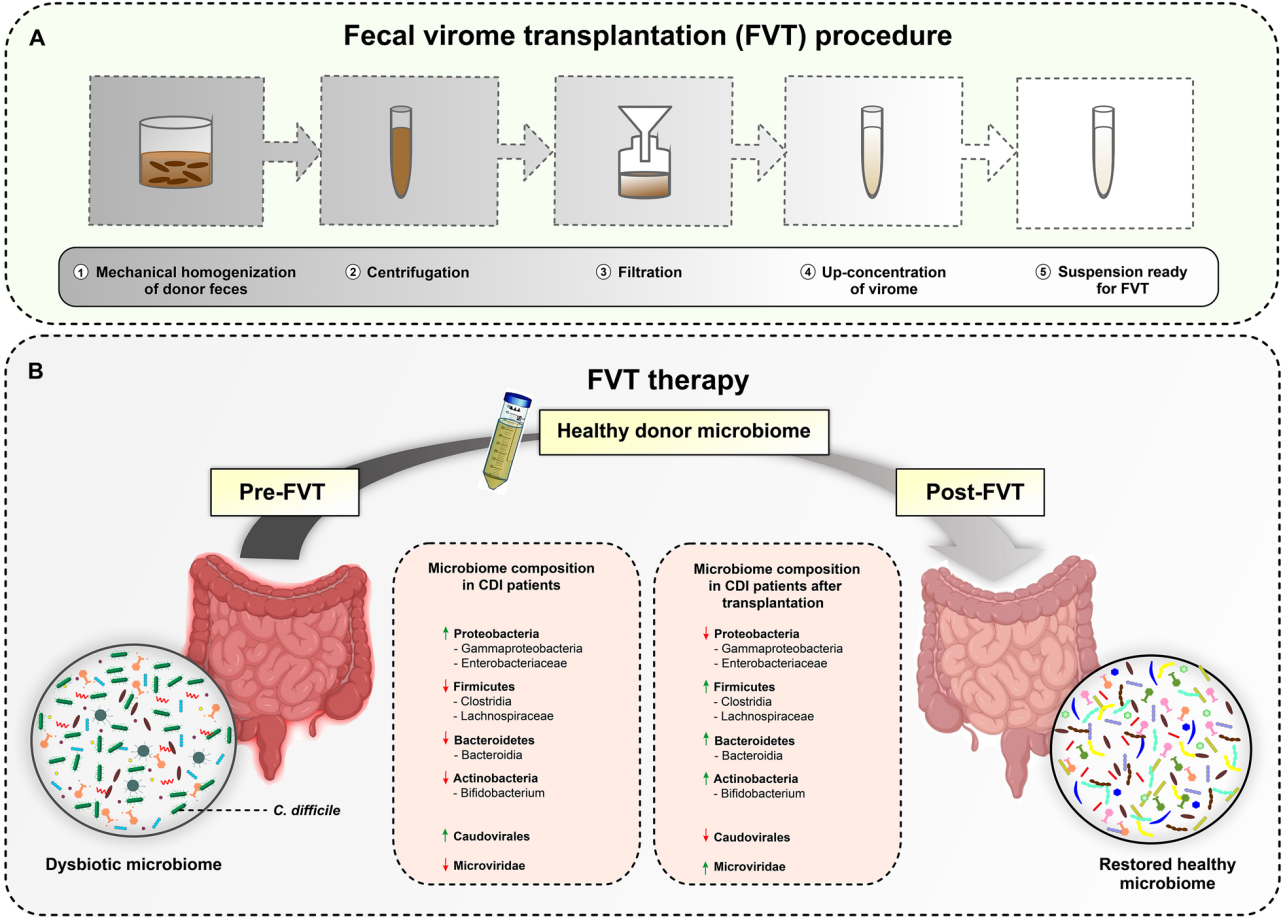
A schematic overview of FVT process. **A** FVT procedure on the fecal sample collected from a healthy donor: (1) homogenization of feces to remove large particles; (2) centrifugation; (3) sterile filtration through a membrane filter for removing bacterial cells and debris; (4) up-concentration with a centriprep centrifugal filter to increase viral titer; (5) the use of concentrated suspension for FVT. **B** Typical alterations in the gut microbiome composition of CDI patients pre- and post- FVT. These changes can result in restoration of healthy and beneficial microbiome. *FVT* fecal virome transplantation, *CDI* *Clostridioides difficile* infection

In addition to refined phage therapy, the use of FVT is getting mounting attention as a new therapeutic option in recent years. So far, FVT has been used for treating a number of diseases linked to gut microbiome dysbiosis such as metabolic syndrome, type 2 diabetes (T2D), and obesity [19, 66]. Although FMT results in an almost complete restoration of the balance of microbiota in dysbiotic patients, the precise mechanism of action of FMT has yet to be fully elucidated [121]. Generally, restoring the recipient’s gut microbiota is achieved by transferring bacteria, phages and other microbes form a healthy donor following FMT [18]. Generally, fecal virus-like particles have a density similar to fecal bacteria, which is about 10^9^ per gram of feces, and more than 90% of the viral density is dominated by phages [122]. Therefore, a large number of fecal phages is transferred during FMT, which may have significant physiological effects on the overall well-being of recipients. Accordingly, some studies demonstrated that bacteriophages play an important role in successful FMT treatments through controlling the disease progression and restoring the balance of the gut microbiome [17, 123]. The procedure of FVT and its routes of administration are almost similar to FMT, albeit it is filtered to exclude intact fecal bacteria. Hence, the use of sterile fecal filtrate of a healthy donor can be administered as a refinement to FMT, which decreases the risk of invasive bacterial infections and adverse events (Fig. 2A). Recent in vivo studies have supported the capability of FVT to normalize the gut microbiota population after antibiotic therapy by affecting both the bacteriome and virome of recipients [13, 19, 124]. More specifically, in a dysbiotic state in CDI patients, a decrease in the abundance of Firmicutes (especially Clostridia), Bacteroidetes, and Actinomycetota, and an increase in the abundance of Proteobacteria (especially Gammaproteobacteria) are commonly observed [17, 125]. After FMT or FVT, the bacterial communities of recipients resemble those of donors, and the abundance of Firmicutes, Bacteroidetes, and Actinomycetota are restored. In addition to bacteriome, the abundance, diversity and richness of virome are also altered in CDI patients, in which a significantly higher *Caudovirales* abundance, lower *Caudovirales* diversity, richness and evenness, and a decreased abundance of *Microviridae* are reported as compared to healthy subjects [17]. Furthermore, it has been shown that FMT treatment resulted in a significant decrease in the abundance of *Caudovirales*, while caused an increase in *Microviridae* abundance (Fig. 2B) [17, 125]. These studies document the effectiveness of FVT treatment in recipients, which is probably due to phage-driven manipulation of the gut microbiota, leading to improved host metabolome and health [18, 19]. Interestingly, a recent study showed that necrotizing enterocolitis (NEC), an inflammatory disease of the small intestine, can be completely controlled by FVT through oral administration in piglets with NEC, whereas ∼42% of cases still showed the disease after administration of FMT [124]. Moreover, these researchers concluded that the application of FVT exhibited higher safety and efficacy than FMT in NEC animal model.

However, there are limited studies characterizing the role of phages in the treatment of CDI or rCDI. Thus, considering the high effectiveness of the FMT to reduce the risk of rCDI, the beneficial role of phages in CDI treatment and their impact on the gut microbiome homeostasis should not be ignored. Furthermore, the utilization of phages would be an innovative approach to combat biofilm formation especially by antibiotic-resistant *C. difficile* strains, which are highly difficult to eradicate using common antibiotic therapy [15, 17, 126].

## Phages and CDI treatment

### Gut phage dynamics during FMT

FMT from healthy donors has acted as a highly efficient microbiome-based therapeutic option for treating rCDI patients with a success rate of more than 90% [127]. However, there are multiple safety issues for the use of FMT in clinical setting, which may limit its widespread application in critically ill or immunocompromised patients [128]. Additionally, due to the lack of a comprehensive and standard donor screening panel, the risk for the transfer of harmful agents from the donor microbiome cannot be fully prohibited [18]. As over mentioned, alterations in the gut microbiota composition after FMT are mostly attributed to the transferred bacterial communities. Based on recent findings obtained from various human microbiome projects, we well know that bacteria are not the only transferred component following microbiota transplants. The non-bacterial gut residents, including viruses, archaea, fungi, protozoa, and parasites, are also transplanted to different sites in the gut of the recipients post-FMT, which play vital roles in the re-establishment of a healthy microbiome [17, 129]. In a recent study by Zuo et al., it was demonstrated that phage compositions are altered in rCDI patients after FMT and resembled to those of the donor, as far as higher *Caudovirales* richness was reported in responders compared with those who did not respond to FMT treatment [17]. They also found that CDI patients demonstrated a significantly lower *Caudovirales* diversity, richness, and evenness compared with healthy household controls, suggesting that *Caudovirales* can play a key role in the success and effectiveness of FMT treatment. It was explained that *Caudovirales* can affect the efficacy of FMT in rCDI patients through directly altering the dysbiotic microbiota or by improving the bacterial colonization. Furthermore, these rCDI patients showed a lower abundance of bacteriophage *Microviridae* compared with healthy subjects. Notably, the enrichment of fifteen viral species of *Microviridae* family was reported in FMT responders. Interestingly, the intestinal phages transferred with FMT can sustainably engraft in the recipient gut microbiota for a protracted period [130]. By analyzing the long-term effects of FMT, it was shown that the phageome composition of rCDI patients who responded to FMT could resemble the phage community of healthy donors and last for 7 to 12 months after treatment [123, 131]. This long-term persistence may be due to the strong adsorption of phages to mucus and epithelial cells in the gut of the recipients [67, 132]. These findings indicate that apart from living bacterial species, other components of the microbiota, particularly bacteriophages, are contributed to the re-establishment of the gut microbiota after FMT treatment [129].

### Phage therapy for CDI treatment

Recently, a great interest has been attracted toward *C. difficile* phages as an alternative to antibiotics for CDI treatment. However, the lysogenic nature of most of the *C. difficile* phages has significantly restricted the application of these viruses for CDI treatment. In addition, most *C. difficile* phages have been recovered after the induction of the host with mitomycin C, while the natural induction of prophages in CDI patients has been reported only in a study conducted by Meessen-Pinard et al. [86]. In this regard, these researchers isolated four *Myoviridae* phages including φMMP01, φMMP02, φMMP03, and φMMP04 from filter- sterilized stool supernatants of CDI patients. This study provides evidence that natural induction of prophage can play a role in killing *C. difficile* cells during episodes of CDI. It is expected that temperate phages can undertake lytic infection, and thus may still be valuable for therapeutic use. So far, different studies have been carried out for the use of temperate phages and phage-derived proteins to treat CDI. A summary of these studies is presented in Table 3.

**Table 3:** Summary of phage therapy studies on *C. difficile*

Type of phage therapy	Phage name	Experiment	Outcome	References
Single-phage therapy	CD140	Hamster	• Phage treatment improved hamster survival • Phage treatment could not protect from a second infection	[133]
	phiCD27	In vitro batch fermentation and human colon models	• Reduction of both vegetative cells, and TcdA and TcdB production from *C. difficile* • Reduction of toxin production by lysogens • No impact on other gut microbes	[102, 134]
	PTLPs derived from *C. difficile* RT078	In vitro	• Reduction of vegetative cells from *C. difficile*	[135]
	phiCDHS1	In vitro	• Reduction of *C. difficile* colonization • Negatively impacts on bacterial pathogenicity, such as downregulation of the regulatory genes involved in metabolism and toxin production	[74, 100]
	CDSH1	In vitro HT-29 tumorigenic colon cell model	• Reduction of *C. difficile* adherence • No cytotoxicity to human cells	[132]
Phage cocktail therapy	phiCDHM1, phiCDHM2, phiCDHM3, phiCDHM4, phiCDHM5, phiCDHM6, phiCDHS1	In vitro and in vivo (hamster model)	• Reduction of vegetative cells from *C. difficile* • Reduction of *C. difficile* colonization, sporulation in hamster model	[74]
	phiCDHM1, phiCDHM2, phiCDHM5, phiCDHM6	*G. mellonella* larvae CDI model	• Reduction and prevention of the biofilm formation in vitro • Phage cocktails were more effective than single phages in preventing biofilm formation	[98]
	phiCDHM1, phiCDHM2, phiCDHM5, phiCDHM6	In vitro batch fermentation model	• Reduction of vegetative cells from *C. difficile* • No impact on other gut microbes like enterobacteria and lactobacilli • Increase in specific commensals, suggesting that phage therapy may protect from further colonization of *C. difficile*	[136]
Endolysin therapy	Endolysin catalytic domain CD27L1–179	In vitro	• Modified endolysin demonstrated greater effectiveness than CD27 • No impact on other gut microbes • Endolysin could be modified to kill other pathogenic species	[137]
	Recombinant protein of catalytic domain of lysin PlyCD (PlyCD1-174)	Ex vivo treatment, mouse colon model	• Reduction of *C. difficile* colonization • Little effect on normal commensal bacteria	[138]
	CD11 and CDG endolysins	In silico and in vitro testing	• Two endolysins were identified from the genomic sequences of *C. difficile* strains	[139]
	Recombinant fusion protein of phiC2 lysin (PlyCD) and human defensin protein HD5	In vitro and in vivo (mouse model)	• MIC of fusion protein was lower than each protein alone • Reduction of *C. difficile* toxin production and sporulation in vivo • Increase in survival rate of mouse model	[140]
	Recombinant protein of CWH lysin and CWH351-656	In vitro and ex vivo	• Hydrolyzing the cell wall of *C. difficile* • Prevention of *C. difficile* spore outgrowth by CWH351-656	[141]
	Endolysin CD16/50L from HN16-1 and f HN50	Homodimer in vivo and in vitro	• Hydrolyzing the cell wall of *C. difficile*	[142]
Engineered phage therapy	Wild-type phiCD24-2, and engineered phiCD24-2 (carrying CRISPR-Cas3 components)	In vitro and in vivo (mouse model)	• Phage modification increased the lytic activity • Modified phages showed higher efficacy for reducing vegetative cells and the bacterial load in feces compared to wild-type parental phages	[143]
FVT	Sterile FFT	rCDI patients	• FFT restored normal stool habits and eliminated symptoms of CDI for a minimum period of 6 months	[18]
	Lyophilized sterile FFT	rCDI patients	• FFT cured 75% of patients and improved the CDI symptoms	[144]

*CDI* *Clostridioides difficile* infection, *FFT* fecal filtrate transplantation, *FVT* fecal virome transplantation, *PTLPs* phage tail-like particles, *rCDI* recurrent *Clostridioides difficile* infection, *RT* ribotype

**Fig. 3:**
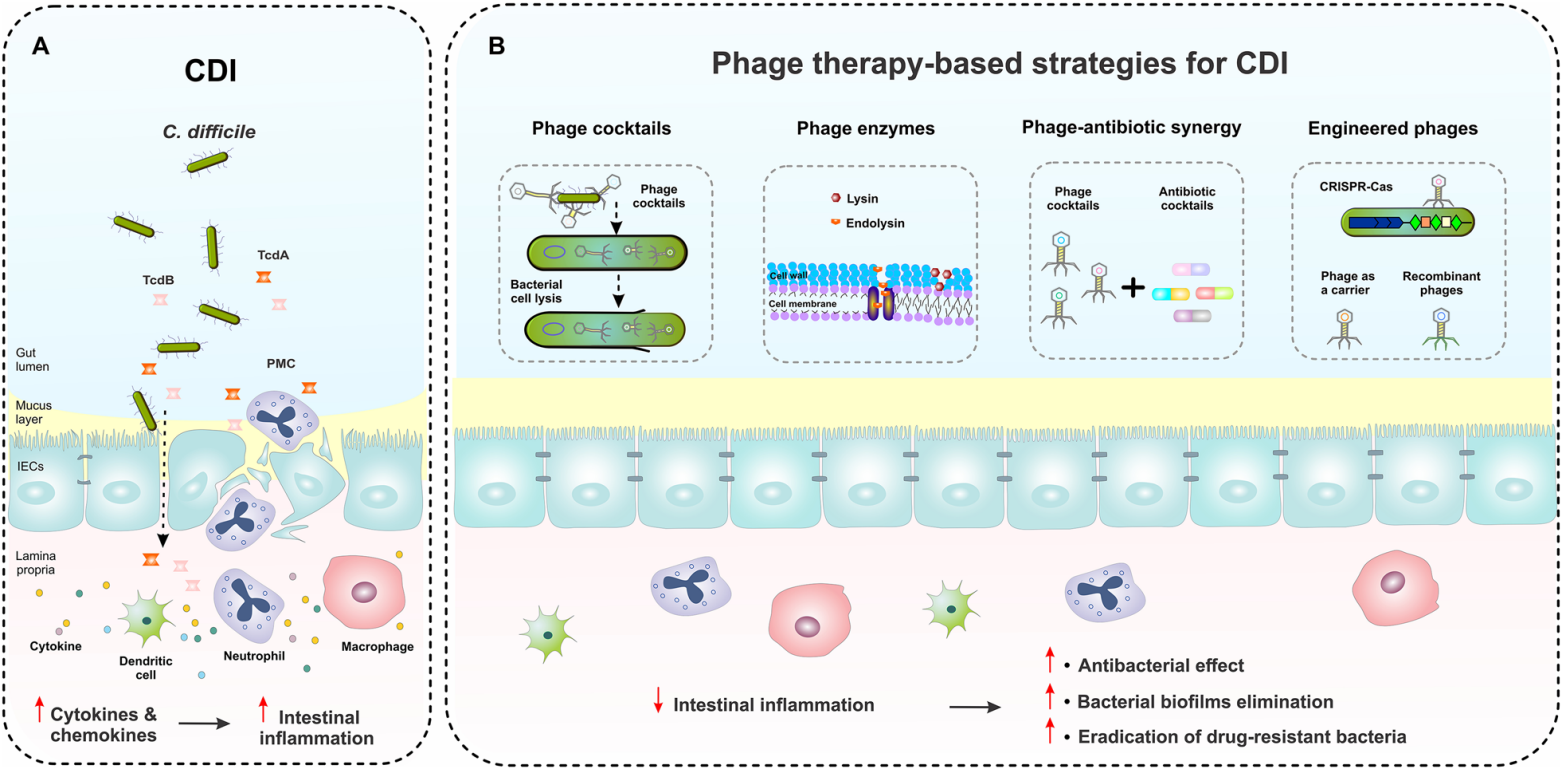
A schematic of *C. difficile* pathogenesis and phage therapy-based strategies for treatment of CDI. **A** In CDI, the gut microbiota dysbiosis increases the susceptibility to *C. difficile* bacteria and toxins. Gut colonization and toxin production by *C. difficile* lead to the disruption of tight junctions and stimulation of immune cell responses through inducing inflammatory cytokine production by neutrophils, macrophages, and dendritic cells, which subsequently can result in intestinal epithelial damage and PMC formation. **B** The application of different strategies of phage-based therapy, including phage cocktails, phage-derived enzymes, the synergy between phages and antibiotics, and phage delivery of CRISPR-Cas system can help control CDI, modulate the intestinal inflammation, and promote the gut homeostasis. *CDI* *Clostridioides difficile* infection, *IECs* intestinal epithelial cells, *PMC* pseudomembranous colitis

Single-phage therapy has been performed as the first phage-based treatment for CDI in 1999. In this regard, the administration of phage CD140 significantly improved the survival of hamsters challenged with *C. difficile* [133]. Furthermore, the use of phage phiCD27 reduced both *C. difficile* vegetative cells and TcdA/TcdB production in batch fermentation and in an artificial gut model [102, 134]. Recently, the therapeutic potential of phage CDHS-1 was indicated, which targets and kills *C. difficile* hypervirulent RT027 strain by reducing its colonization and applying negative impacts on bacterial pathogenicity [100]. Interestingly, this phage can act more effectively in the presence of epithelial cells than when used to infect bacterial cells alone [132]. Therefore, this finding suggests that CDHS-1 has promising therapeutic potential for controlling the infection in the gut. However, there is little information about the mode of action of CDHS-1 for modulating its bacterial host genome during the infection cycle, thus further investigations are needed to help ascertain the potential therapeutic implications of this phage in the future. Moreover, single-phage therapy has some shortcomings due to the lysogenic nature of phages, their narrow host spectrum, and the emergence of phage resistance [74]. Hence, the use of phage cocktails, combination of phage and antibiotics, phage-derived enzymes, and phage engineering can be a superior strategy to overcome the pitfalls of single-phage therapy for CDI treatment (Fig. 3) [74, 98].

The effectiveness of the phage cocktail strategy to treat CDI has been investigated in both in vitro and in vivo experiments [74, 100, 136, 145]. Nale et al. examined the effectiveness of temperate phages phiCDHM1 to phiCDHM6 as an individual or cocktail against different strains of *C. difficile* [74]. Based on their results, multiple-phage cocktails, especially combinations including phiCDHM 1, 2, 4, and 6, could kill a broader range of *C. difficile* strains without regrowth than a single phage. Interestingly, there was increased phage resistance against individual phages, whereas phage resistance was limited in the application of phage cocktails. In another study, Nale et al. demonstrated that a 4-phage cocktail targeting *C. difficile* could reduce and prevent biofilm formation of *C. difficile* RT014/020 or also eliminate the bacteria [98]. The effectiveness of multiple-phage cocktails was further investigated in an in vivo model and it was shown that the use of a combination of phiCDHM 1, 2, 5, and 6 can reduce the number of spores in the cecum and colon of CDI hamsters [74]. Most importantly, the abundance of commensals, including total anaerobes, lactobacilli, and enterobacteria, was not altered by phage therapy. This suggests that phage-based treatment can disturb the microbiome structure, however, further studies are required to validate this claim.

As aforementioned, the use of phage lysins has also been proposed to overcome limitations of the lysogenic nature of phages. Endolysin is a peptidoglycan hydrolase that is encoded by lytic phages. Endolysin helps releasing of phage progeny by disrupting bacterial cell wall during the final step of viral infection. The phiCD27 endolysin (CD27L) is the first endolysin characterized in *C. difficile*, which has exhibited promising effect in controlling a panel of *C. difficile* strains over a wide range of pH conditions [40, 83, 137]. These results show that the application of phage lysins can provide specific treatment options for CDI, in particular for drug-resistant *C. difficile* strains [146]. Notably, the partial sequence of N-terminal portion of CD27L, and CD27L1-179, exhibited a broader lytic range than the full length itself. Also, both CD27L and CD27L1-179 were harmless to other gut commensal microorganisms [137]. PlyCD is another endolysin derived from a prophage of *C. difficile* 630, which shows strong lytic activity against a variety of *C. difficile* strains. The catalytic domain of N-terminal portion of PlyCD (PlyCD1-174) displayed higher lytic activity than the full-length [138]. A recent study demonstrated that the expression of a recombinant fusion protein containing the catalytic domain of the endolysin from phiC2, and the functional domain of the human defensin protein HD5 can help enhance the effectiveness of endolysin for CDI treatment by reducing sporulation and toxin production [140]. Additionally, the use of this fusion protein showed a lower minimum inhibitory concentration (MIC) than conventional antibiotics in vitro. Therefore, the use of genetic engineering method would be a valuable tool to improve the function of endolysins and reduce the risk of bacterial resistance.

Recently, CRISPR-Cas gene editing systems have been applied for the genetic engineering of bacteriophages. In this regard, phiCD24-2 was engineered to bear a genome that targets CRISPR-Cas3 found in *C. difficile*, and successfully converted lysogenic phages to lytic forms [143]. Although there was no difference between the engineered phage and wild-type phage in terms of host range or phage morphology, the engineered phage showed higher efficiency in controlling *C. difficile* than wild-type phage in both in vitro and in vivo models, indicating the superiority of the engineered phage for CDI treatment.

In addition to bacteriophages, PTLPs are a promising and potential alternative therapy for treating CDI. PTLPs are morphologically similar to bacteriophages, and thus may have a bacteriophage origin. Although there is no evidence for existing viral genome in PTLPs, they can kill their bacterial hosts and prevent the release of bacterial toxins. The specific lytic activity of PTLPs against *C. difficile* has been investigated in some studies [135, 147]. For example, Sangster et al. demonstrated that PTLPs isolated from the *C. difficile* RT078 strain can lyse various RT027 isolates, but did not display activity against other strains [135]. Therefore, PTLPs may have a broader host range than phages, but a narrower host range than endolysins. However, further research is necessary in order to apply any of these therapeutic tools in clinical practice.

### FVT for CDI treatment

The application of FVT for rCDI treatment has been considered in recent years. Recently, some studies have shown that treating rCDI with sterile fecal filtrate obtained from a healthy donor can alleviate the symptoms of rCDI. A pilot study successfully treated 5 of 5 rCDI patients using FVT [18]. Another study demonstrated that the use of lyophilized sterile filtrate and lyophilized donor stool can successfully help the treatment of 75% (3/4) and 80% (4/5) of the rCDI patients, respectively [144]. These results suggest that the phageome composition can play a key role in restoring the gut microbiota following FVT, and can be considered as a safer refinement than FMT [18, 19, 124]. Additionally, the application of mixed virome from several healthy donors may increase the effectiveness of FVT because of targeting a larger fraction of the recipient gut bacteria than a single virome [19]. However, the current understanding of the virome community transferred by FVT and insights about the precise interactions of these components with the gut microbiota are limited and require additional in-depth studies.

## Discussion

There is an obvious need for developing new alternative therapeutic approaches to conventional antibiotic-based treatments for infectious diseases such as CDI, by which the integrity and essential functions within gut microbiota are maintained. Today, phages are widely recommended to be applied as an efficient tool to help modify the gut microbiota composition without causing substantial disruptions to the overall microbial community structure [19]. The efficacy of phage therapy to treat many infectious diseases, especially in combination with traditional antibiotics, has been reported in recent years, which highlights its potential as a promising strategy to overcome antibiotic-resistant infections [126].

However, the current knowledge regarding the use of phage therapy for the management of CDI is in its infancy. Till now, several *C. difficile* phages have been identified, although none of them have been fully characterized [148]. Most isolated *C. difficile* phages are temperate, which can remain as a prophage in the genome of the infected bacterial hosts, and influence bacterial virulence, such as production of toxins and formation of biofilms [65]. The lysogenic nature of the *C. difficile* phages is an important challenge for their use as therapeutic agents, however, temperate phages are not precluded from the lytic life cycle and may switch to a lytic pathway as well. The efficacy of single-phage therapy for the treatment of CDI has been evaluated in multiple studies. Given that single-phage therapy could provide the possibility of emerging phage resistance mostly due to phage DNA breaking by CRISPR-Cas systems, phage receptor mutations, or through superinfection exclusion by temperate phages [101], applying this approach may encounter critical obstacles for use in clinical practice. One solution for this disadvantage is the use of a combination of various phages, which can demonstrate a synergism in antimicrobial activity by multiple phages attacking the same bacterial cell, and also limit bacterial evolution of phage resistance [149]. Recently, the administration of optimized phage cocktails was found to inhibit *C. difficile* growth and reduce *C. difficile* colonization using in vivo and in vitro models [74, 136]. Additionally, phages or phage-derived enzymes can affect both antibiotic-sensitive and antibiotic-resistant *C. difficile* strains, and can be administered as supplements in combination with antibiotics, which may reduce the possibility of emerging bacterial resistance and enhance the antibacterial effect [14, 113, 150].

Moreover, recent genomic discoveries and progress in genetic engineering allow us to overcome the lysogenic nature of *C. difficile* phages through the construction of desired therapeutic phages [148]. Additional genetic manipulation can help increase the antimicrobial activity of CRISPR-enhanced phages by modulating toxin expression in the bacterial host. Therefore, genetic engineering can not only be exploited to mitigate the problems related to phage lysogeny but also diminish the expression of virulence attributes of pathogenic agents.

It has been documented that FVT can cause shifting the gut microbiota structure to a steady state and restore hemostasis in CDI patients [18, 144]. Additionally, lytic phages with a broad host range are able to infect *C. difficile* cells and thereby inhibit their growth after a successful FVT [17, 19, 46, 123]. Studies have reported that increased diversity, richness, and evenness of *Caudovirales* were associated with the efficacy of FVT or FMT in CDI [17]. However, there has been an increase in the richness of *Caudovirales* in other intestinal diseases like IBD, indicating that a vast expansion of *Caudovirales* bacteriophages may contribute to intestinal inflammation and bacterial dysbiosis through decreasing bacterial richness and diversity [17, 43, 151]. Several studies have shown that the administered phage titer is an essential factor for the efficacy of phage-based therapeutics [152–154]. For example, the use of phage titers ranging from 10^8^ to 10^10^ PFU/mL resulted in higher efficiency of phage therapy for treatment of various infectious bacterial diseases [74, 152, 153]. However, further functional studies on the virome of healthy donors and transplant responders are required to elucidate the precise role of phages in the initiation of a cascade of events that ultimately helps normalize the gut microbiota composition.

## Conclusion

The therapeutic and antibacterial application of strictly lytic or virulent bacteriophage viruses to cure various microbial infections, known as phage therapy, has been recognized for more than a century. In recent years, and in view of the rising number of reports available, phage therapy emerges to be a promising alternative option in the treatment of *C. difficile -*related infections, in particular recurrent and drug-resistant infections. Moreover, the potential applications of phage therapy make it utterly conceivable to be exploited at least in some cases instead of antibiotics for patients with eradication failure, while limiting the development of new refractory hypervirulent strains by reducing the administration of antibiotics. The potential application of phages in manipulating the dysbiotic gut microbiota composition to a homeostatic state is also becoming an area of intense research focus. However, due to the lack of sufficient data on the biology, dynamic, evolutionary events, phage-host interactions, clinical safety and efficacy of *C. difficile*- specific phages, great attention should be taken in consideration for applying phage therapy such as FVT against CDI. Further research is definitely required in this field to meet these various scientific and technical bottlenecks and challenges in the development of phage therapy for the treatment of CDI patients, and also for animal health, the environment, and the beyond.

## Data Availability

Not applicable.

## Abbreviations

ACG: American College of Gastroenterology
ADPRTs: Adenosine-diphosphate-ribosyltransferases
ANKp: Ankyrin protein
ARGs: Antibiotic-resistant genes
*C. difficile*: *Clostridioides difficile*
CDI: *C. difficile* infection
CDT: *C. difficile* binary toxin
CRISPR/Cas9: Clustered regularly interspaced short palindromic repeats/CRISPR-associated protein 9
CRC: Colorectal cancer
CWPs: Cell wall proteins
dsDNA: Double-stranded DNA
FDA: Food and Drug Administration
FFT: Fecal filtrate transplantation
FMT: Fecal microbiota transplantation
FVT: Fecal virome transplantation
GI tract: Gastrointestinal tract
HGT: Horizontal gene transfer
IBD: Inflammatory bowel disease
ICTV: International Committee for Taxonomy of Viruses
IECs: Intestinal epithelial cells
MGEs: Mobile genetic elements
MIC: Minimum inhibitory concentration
NEC: Necrotizing enterocolitis
PaLoc: Pathogenicity locus
PMC: Pseudomembranous colitis
PTLPs: Phage tail-like particles
QS: Quorum sensing
RBPs: Receptor-binding proteins
rCDI: Recurrent *C. difficile* infection
RT: Ribotype
SARS-CoV-2: Severe acute respiratory syndrome coronavirus 2
SCFAs: Short-chain fatty acids
ssDNA: Single-stranded DNA
SlpA: Surface layer (S-layer) protein A
T2D: Type 2 diabetes
TcdA: Toxin A
TcdB: Toxin B
